# Catestatin peptide impedes melanoma progression and drug resistance by reprogramming oncogenic signaling pathways

**DOI:** 10.21203/rs.3.rs-8548714/v1

**Published:** 2026-01-19

**Authors:** Sushil Mahata, Satadeepa Kal, Suborno Jati, Kechun Tang, Nicholas Webster, Angelo Corti

**Affiliations:** University of California San Diego and VA San Diego Healthcare System; VA San Diego Healthcare System; VA San Diego Healthcare System; VA San Diego Healthcare System; VA San Diego Healthcare System

**Keywords:** Catestatin, melanoma, peptide therapy, drug resistance reversal

## Abstract

Melanoma remains one of the most aggressive and treatment-resistant cancers, emphasizing the need for novel therapeutics. In our current study we report a peptide-based approach as potential therapeutic. Here we report for the first time the involvement of Catestatin (CST) peptide in carcinogenesis, with melanoma identified as unexplored and therapeutically relevant context. The expression and role of CST, a Chromogranin A (CgA)-derived peptide with immunomodulatory and reparative properties in skin injury led us to examine its connection to melanoma. Advancing melanoma stages showed decreased CST expression. CST administration to patient derived cells and melanoma cell lines A375, B16F10, SKMEL28 revealed increased apoptosis, decreased proliferation and metastatic ability of the melanoma cells without affecting cell viability for normal skin fibroblasts. CST reduced growth kinetics and tumor weight in B16F10 derived in vivo melanoma tumors. Transcriptomic analyses of CST-treated human cell line and mouse tumor revealed downregulation of hypoxia, collagen remodeling, epithelial to mesenchymal transition pathways (EMT), stress adaptive responses that are accountable for melanoma progression. In Vemurafenib-resistant A375 cells, CST increased apoptosis and repressed multiple resistance associated genes. These results highlight CST as a promising therapeutic candidate capable of opposing melanoma progression and overcoming resistance to current targeted treatments.

## 1. INTRODUCTION

Melanoma is an escalating cancer worldwide, with incidences rising faster than any other solid tumor over recent decades (1). Although melanoma represents a small fraction of skin cancers, it accounts for over 80% of skin cancer–related deaths owing to its aggressive metastatic potential and resistance to therapy (1). The advent of targeted therapies - particularly BRAF and MEK inhibitors - and immune checkpoint blockade has substantially improved outcomes for patients with advanced disease (2, 3). Small-molecule inhibitors Dabrafenib and Vemurafenib, which selectively target *BRAF^V600E* mutations, are now standard of care for a majority of melanoma patients (3, 4), whereas those harboring *KIT* mutations benefit from Imatinib (5). Moreover, advent of immune checkpoint inhibitors has revolutionized melanoma management, extending median survival from approximately six months to nearly six years (6).

Despite breakthroughs, multitude of patients either fail to respond or acquire resistance during treatment, resulting in relapse and mortality (7, 8). These limitations highlight the urgent need for novel therapeutic approaches engaging mechanisms beyond the current ones.

Chromogranin A (CgA), a prohormone abundantly expressed in neuroendocrine tissues (9, 10), undergoes proteolytic processing generating multiple biologically active peptides. Among them, Catestatin (CST; hCgA_352-372_) emerged as a multifunctional regulator of cardiovascular, metabolic, and immune homeostasis (11–15). CST exerts protective effects, including anti-inflammatory (16, 17), antihypertensive (18–22), and anti-diabetic (16, 23) actions. It inhibits catecholamine release (24–26), regulates endothelial proliferation and migration (17, 27, 28), and modulates macrophage and lymphocyte phenotypes (16), supporting its role as a systemic homeostatic peptide with therapeutic potential.

CST is expressed in human skin, with increased levels following injury (29), and exerts functional activity in keratinocytes (30). CST-like immunoreactivity has been reported in rodent skin and sensory ganglia (31), suggesting a conserved cutaneous role. However, its relevance in oncogenic progression has not been explored.

In the present study, we identify CST as a previously unrecognized regulator of melanoma progression in both human and mouse models. We demonstrate that CST expression decreases with advancing melanoma and exogenous CST administration markedly suppresses melanoma cell viability, proliferation, and migration across patient-derived and established cell lines. In syngeneic B16F10 tumor models, CST administration significantly reduces tumor growth and enhances apoptosis, confirming its anti-tumorigenicity *in vivo*. Transcriptomic analyses reveal that CST downregulates key pathways associated with extracellular-matrix organization, hypoxia response, and collagen metabolism - hallmarks of melanoma invasion and metastasis. Notably, CST suppresses several resistance-associated genes, including *FGFR3, PDGFRB, ID1/2/3, SREBF1*, and *MITF*, in Vemurafenib-resistant A375 cells, suggesting a role in overcoming therapeutic resistance.

Collectively, our findings establish CST as a novel melanoma-regulatory peptide with multifaceted antitumor activity. By integrating clinical, molecular, and *in vivo* evidence, this study positions CST as a promising therapeutic candidate. Given its pleiotropic regulatory properties and favorable safety profile, CST may inaugurate a new class of peptide-based therapeutics that modulate oncogenic pathways to inhibit melanoma progression and resistance.

## 2. MATERIALS AND METHODS

### 2.1 Cell Culture

Mouse embryonic fibroblast (MEF), human skin fibroblast (CCD1076), human melanoma cell lines (A375, SK-MEL-28), and mouse melanoma cell line (B16F10) were obtained from the American Type Culture Collection (ATCC). Cells were maintained in DMEM supplemented with 10% fetal bovine serum (FBS), 100 U/mL penicillin, and 100 μg/mL streptomycin in a humidified incubator at 37°C with 5% CO_2_.

Patient-derived melanoma cell lines (K06184, 156681, and 128128-mixed tumor cultures) were procured from the NCI Patient-Derived Models Repository (PDMR) and cultured following PDMR standard operating procedures in DMEM/F-12 containing 5% FBS, 0.4 μg/mL hydrocortisone, 0.01 μg/mL epidermal growth factor, 24 μg/mL adenine, 100 μg/mL penicillin-streptomycin, 2 mM L-glutamine, and 10 nM Y-27632 dihydrochloride on Matrigel-coated plates. Details of patient derived melanoma cells can be obtained from https://pdmdb.cancer.gov/web/apex/f?p=101:41 and a short description from Supplementary Table 1. All cells were checked for mycoplasma using Universal mycoplasma detection kit from ATCC (Catalog #30-1012K)

### 2.2 Human Tissue Samples

Commercially available human melanoma tissue microarrays (catalog nos. Me482A and Me551) were purchased from TissueArray.com for immunohistochemical analyses.

### 2.3 Peptide Treatment

Catestatin (CST; sequence SSMKLSFRARAYGFRGPGPQL) was synthesized by GenScript and dissolved in 0.9% saline. Cells were treated with CST at concentrations of 0.5–10 μM as indicated. For *in vivo* studies, CST was administered intraperitoneally at 10 mg/kg body weight, three times per week (Monday, Wednesday, Friday).

### 2.4 CST cellular uptake assay

Biotinylated CST synthesized from GenScript was added to A375 cells; 5 μM for 45 minutes. Cells were fixed in paraformaldehyde and AF488 tagged Streptavidin (catalog #S32354) and Hoechst was added in permeabilized cells for 1 hour. Cells were washed in PBS and mounted with Fluoromount G (Thermo Scientific) and observed in 60X oil immersion lens in a Nikon AXR Confocal, NSPARC super resolution microscope in UCSD core.

### 2.5 Bioinformatic Analyses

Expression of CCN2, LOXL2, DDIT4, and PDGFRB in primary and metastatic skin cutaneous melanoma samples was analyzed using the UALCAN database (http://ualcan.path.uab.edu) (69).

### 2.6 Caspase Assay

Cells (1 × 10^4^ per well) were seeded in 96-well white plates and treated with or without CST for 48 h (A375, SK-MEL-28, B16-F10) or 120 h (patient-derived lines K06184, 128128, 156681). Caspase-3/7 activity was measured using the Caspase-Glo^®^ 3/7 Assay System (Promega-G8090) following the manufacturer’s protocol. Luminescence was recorded using a Varioskan LUX microplate reader (Thermo Fisher Scientific).

### 2.7 Live/Dead Viability Assay

Cells treated with CST or vehicle for 120 h, were incubated with 2 μM calcein AM and 4 μM ethidium homodimer III (Biotium Viability/Cytotoxicity Assay Kit #30002) for 30 min at room temperature in the dark. After washing, cells were imaged under FITC and Texas Red^®^ channels using a Keyence fluorescence microscope (20×).

### 2.8 Cell Viability Assay

Cells (1 × 10^4^ per well) were plated in 96-well plates and treated with CST (0.5–10 μM) for 72–120 h. Viability was determined using the CCK-8 reagent (APExBIO-K1018). Absorbance was measured at 450 nm using a Varioskan LUX reader.

### 2.9 Colony Formation Assay

A375, SKMEL28, and B16F10 cells (1 × 10^3^ per well) were treated with or without CST according to the experimental design and cultured for 15 days. Colonies were fixed in methanol for 20 min, stained with 0.1% crystal violet, and photographed.

### 2.10 Transwell Migration Assay

Equal number of cells were seeded in serum-free medium in transwell inserts (8 μm pore size) and were treated with CST or control for 48h. The lower chamber contained complete medium. After 48 h, non-migrated cells were removed, membranes were fixed, stained with crystal violet, and imaged using a Keyence brightfield microscope (20×) as described previously (70).

### 2.11 Wound Healing Assay

A375 cells at approximately 50% confluency was scratched with a sterile pipette tip, followed by CST or control treatment. Images were captured at 0 and 24 h using brightfield microscopy (20×).

### 2.12 RNA Sequencing

Total RNA from mice tumor and A375 cell line was isolated using RNeasy miniprep Kit (Qiagen). RNA was quantified by Nanodrop spectrophotometer and integrity was evaluated by Tapestation (Agilent). Complementary DNA library was prepared using 400ng of RNA using mRNA HyperPrep Kit (KAPA) according to manufacturer’s protocol with Unique Dual-Indexed adapters (KAPA). The cDNA library was amplified and assessed by Qubit2.0 (Thermo Fisher Scientific). The libraries were then pooled and sequencing was done in NovaSeq X Plus 10B (Illumina) in UCSD IGM core (71).

### 2.13 RNA Sequencing analysis

Raw RNA-seq reads were quantified using Salmon (v1.10.3) in quasi-mapping mode against the Homo sapiens or Mus musculus reference transcriptome (Human: Ensembl Release GRCh38.115, Mouse: Ensembl Release GRCm39.115). Transcript-level abundance estimates (quant.sf files) were generated for each sample.

Transcript-to-gene mapping was obtained from the relevant Ensembl GTF annotation file using the GenomicFeatures and AnnotationDbi R packages. Transcript quantifications were imported and summarized at the gene level with tximport (v1.36.1), setting ignoreTxVersion = TRUE. The resulting gene-level count matrix served as the basis for further analysis.

#### Differential expression analysis (DESeq2)

Gene expression differences between treatment groups were assessed using DESeq2 (v1.48.1) in R. The workflow included normalization with size factors, dispersion estimation, and Wald tests, following the default DESeq2 procedures. Genes with an adjusted p-value (Benjamini - Hochberg FDR) < 0.05 and |log_2_-fold change| > 1 were considered significantly differentially expressed.

#### Annotation and visualization

Ensembl gene IDs were mapped to gene symbols using the org.Hs.eg.db (v3.21.0, for Human) or org.Mm.eg.db (v3.21.0, for Mouse) annotation packages. Volcano plots, generated with ggplot2 (v4.0.0), highlighted significantly up- and down-regulated genes. All analyses were based on DESeq2 results. Gene expression heatmaps were created using the Complex Heatmap package (v2.24.1) in R, based on Log2-transformed normalized counts. GO Biological Process Enrichment analysis of the up- and down-regulated genes was conducted with clusterProfiler (v4.16.0) and shown as bar plots. Common genes between mouse and human transcriptomic analyses were identified using the VennDiagram package (v1.7.3) in R.

### 2.14 Generation of Vemurafenib-Resistant Cells

Vemurafenib-resistant A375 cells were generated following established protocol. Parental cells were treated with 100 nM Vemurafenib (SelleckChem #S1267) for 2 weeks, followed by stepwise doubling of drug concentration every 2 weeks up to 3.2 μM. Surviving cells in maintenance culture containing 2 μM drug was used for downstream studies.

### 2.15 Mouse Tumor Model

Age-matched male and female *C57BL/6* mice (7–9 weeks, n = 9) were subcutaneously injected with 5 × 10^4^ B16-F10 cells in 100 μL PBS. Ten days post-injection, CST (10 mg/kg) was administered intraperitoneally three times weekly. Tumor dimensions were measured with calipers, and volume was calculated using the formula V = (length × width^2^)/2. Mice were euthanized 22 days post-inoculation by inhalational isoflurane that follow the institutional IACUC protocol and AVMA guideline for Euthanasia of animals (2020). Animals were kept in an induction chamber containing 3–5% isoflurane in oxygen which had a flowrate of 1-2L/min. Animals were continuously monitored till the loss of righting reflex and response to toe pinch was absent, thus confirming a surgical plane of anesthesia. Tissues were collected when the animals went into deep anesthesia and euthanasia was finally completely by exsanguination. All procedures were approved by the IACUC of UCSD and the VA San Diego Healthcare System and conformed to NIH guidelines.

### 2.16 Immunohistochemistry and Histology

Fixed tumors were paraffin-embedded and sectioned (5 μm). Hematoxylin and eosin staining was performed at the La Jolla Institute for Immunology. Immunohistochemistry was carried out using the Super Sensitive^™^ M Polymer-HRP Kit (Biogenex). After deparaffinization, antigen retrieval was performed using Histo VT (Nacalai) at 90°C for 20 min. Sections were incubated overnight at 4°C with anti–Ki-67 (Abcam-16667), anti- CCN2,anti-LOXL2 ,anti-DDIT4(Proteintech-25474-1-AP; 15232-1-AP; 10638-1-AP) anti-PDGFRB(Cell Signaling Technology-3169T) or anti-CST (gift from Angelo Corti) at 1:100 dilution. Detection was performed with DAB and counterstained with hematoxylin. Slides were dehydrated and mounted in DPX. Images were captured using Keyence brightfield microscopy (20×).

### 2.17 TUNEL Assay

Apoptotic nuclei were detected in tumor sections using the CF^®^594 TUNEL Assay Kit (Biotium #30064). After deparaffinization and rehydration, sections were permeabilized with 20 μg/mL Proteinase K for 1 h at 37°C, incubated with TUNEL reaction mix for 2 h, counterstained with Hoechst, and mounted in antifade medium. Fluorescence images were obtained using a Keyence microscope.

### 2.18 Phase-Contrast Microscopy

Morphological alterations in CST-treated A375 and Vemurafenib-resistant A375 cells were assessed using Keyence phase-contrast microscopy.

### 2.19 Western Blotting

Equal protein amounts (10 μg) from tumor lysates prepared in RIPA buffer containing protease inhibitors (Thermo Fisher) were subjected to western blotting and incubated with antibodies against caspase-3 (Proteintech-19677-1-AP) and β-actin (Cell Signaling Technology #4970S). For A375 cell line, equal amount (15μg) of con and CST treated lysates were subjected to western blotting. They were probed using antibodies against DDIT4, LOXL2, CCN2 (Proteintech 10638-1-AP; 15232-1-AP ;25474-1-AP) and caspase-3 (Proteintech 19677-1-AP) HRP-conjugated secondary antibodies (Cell Signaling Technology 7076S,7074S) were used, and signals detected by chemiluminescence. Densitometric analyses were performed using Biorad Image Lab software.

### 2.20 *In vitro* plasma stability and *in vivo* plasma pharmacokinetics

Plasma stability was determined in 5 μg CST/ml spiked mouse (C57BL/6) plasma (50 μl) and taking aliquots at specific times over a 24-hour period. Each aliquot (50 μl) was mixed with 150 μl methanol, vortexed, spun, removed the supernatant and reconstituted the pellet with 150 μl 10% formic acid in water. The sample was vortexed and spun and a 100 μl aliquot was taken, diluted 1:1 with 10 mM ammonium bicarbonate, and analyzed by LC-MS/MS. Using the remaining vs. time dataset, we performed a first-order decay analysis by fitting a linear regression to the natural log-transformed percent remaining values (excluding 0% and t = 0 anchor point) to plot the graph.

For *in vivo* plasma pharmacokinetics, male C57BL/6 mouse received a 5mg/kg intraperitoneal dose of CST. Blood samples (100 μl) were obtained at 0, 0.25, 0.5, 1, 2, 4, 8, and 24 hours via tail vein bleeds. Plasma concentrations of CST were determined by LC-MS/MS methods as described above.

### 2.21 Statistical Analyses

Data were analyzed using GraphPad Prism 10. Two-tailed Welch’s *t*-test was used for single-variable comparisons, and one-way or two-way ANOVA for multivariate datasets (e.g., concentration and time). Statistical significance was defined as *p ≤ 0.05, **p ≤ 0.01, ***p ≤ 0.001, ***p ≤ 0.0001 and non-significant as NS.

## 3. RESULTS

### 3.1 Catestatin levels decline with advancing melanoma and exhibit anti-proliferative effects on patient-derived melanoma cells

**Figure 1:**
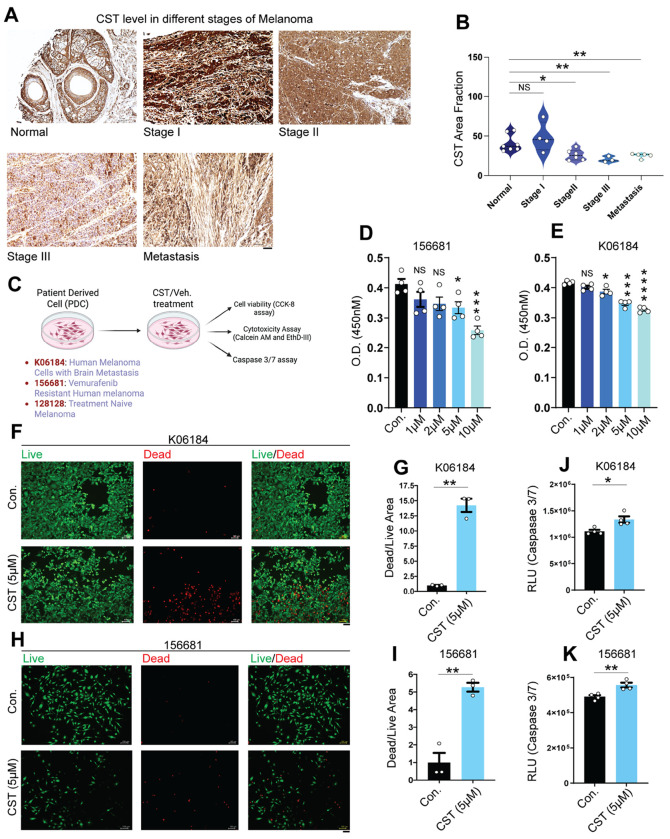
Decline in endogenous CST levels in human melanoma samples and melanoma cell proliferation inhibition upon extraneous CST treatment **(A)** Representative immunohistochemistry images showing levels of Catestatin (CST) in different melanoma stages (normal, stage I, stage II, stage III, and metastasis) in human samples from TissueArray.com. **(B)** Quantification of the immunoreactive CST as area fraction as determined by Fiji3 software. (**C)** Schematic for experimental setup on melanoma patient derived cells (K06184, 156681, and 128128). (**D&E)** Cell viability assay performed on patient derived cell 156681 and K06184 for 120 hours in response to different concentrations of CST (1 μM, 2 μM, 5 μM, and 10 μM as compared to vehicle control (n=4). (**F&H)** Live and dead cell imaging of mammalian cells microscopy showing calcein AM (green) stained live cells and EthD-III (red) stained dead cells upon CST treatment versus control in K06184 and 156681 cells. Images were captured in Keyence microscope at a magnification of 10X. Scale bar for imaging is 100 μm. (**G&I)** Dead/Live area fraction measured upon CST treatment (5 μM) in K06184 and 56681 cells (n=3). (**J&K)** Caspase 3/7 assay done in CST-treated and untreated K06184 and 156681 cells. (n=4). Data were presented as Mean ± SEM. Statistical analyses were done using one-way ANOVA (D&E) and Welch’s t-test (B,G,J,I,K). *p ≤ 0.05, **p ≤ 0.01, ***p ≤ 0.001, ****p ≤ 0.0001.

CST has been detected in human skin (29) and keratinocytes (30) as well as in rodent skin and ganglia (31), but its presence in melanoma patient samples remained unassessed. To address this, we analyzed CST protein expression across progressive stages of melanoma using tissue microarray slides (TissueArray.com). Immunohistochemical (IHC) analysis revealed a marked decline in CST expression with advancing melanoma stages, whereas normal skin and stage I melanoma tissues retained high CST levels (Fig. 1A). Quantitative analysis confirmed a significant reduction in CST staining intensity as melanoma progressed (Fig. 1B).

To determine whether CST administration affects melanoma cell viability, we used patient-derived melanoma cell lines K06184, 156681, 128128 (characteristics mentioned in **Supplementary Table 1**) which were mixed tumor cultures; from the NCI-PDMR repository and normal human dermal fibroblasts (CCD1076). A schematic of the experimental plan with the patient derived cell lines are mentioned (Fig. 1C). CST treatment (1 μM, 2 μM, 5 μM, 10 μM) for 120 hours caused dose-dependent reduction in melanoma cell viability (Fig. 1D&E; **Supplementary Fig. S1B**), whereas normal fibroblasts remained unaffected even at the highest concentration (**Supplementary Fig. S1A**). Viability assays showed elevated dead cells (EthD-III, red) relative to live cells (Calcein-AM, green) following CST treatment (Fig. 1F–H; **Supplementary Fig. S1C**). Quantification revealed a significantly elevated dead-to-live cell ratio (Fig. 1G–I, **Supplementary Fig. S1D**). Corroboration of these results by increased caspase-3/7 activity confirm induction of apoptosis (Fig. 1J–K, **Supplementary Fig. S1E**), and hint at a possible role of CST in curbing melanoma progression.

### 3.2 Catestatin translocates to melanoma cells and reduces viability, proliferation, and migration in melanoma cell lines

**Figure 2:**
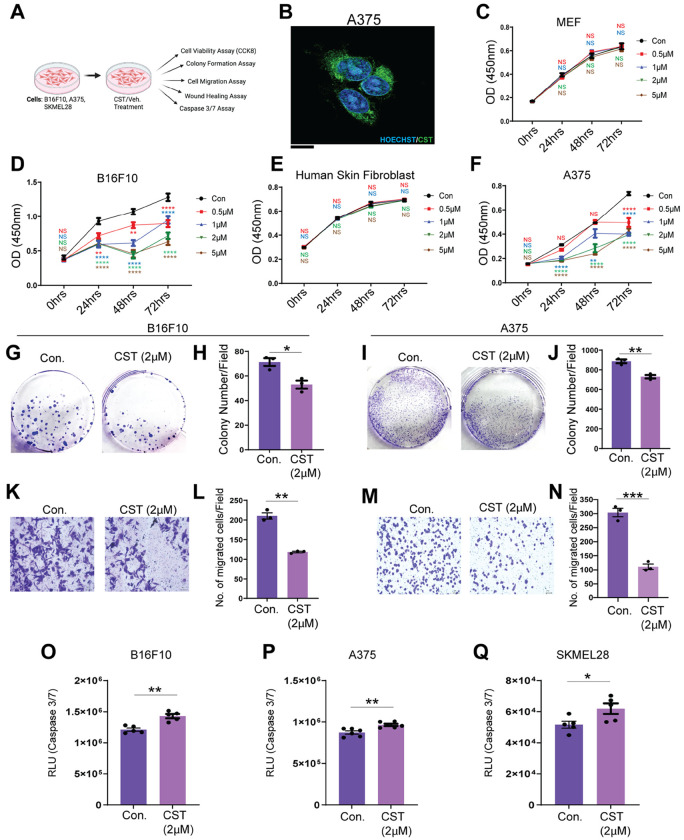
Melanoma progression inhibition upon CST treatment in mouse and human melanoma cell lines **(A)** Schematic representation of experimental setup on B16F10, A375 and SKMEL28 cell line in control and CST-treated condition. (**B**) Confocal imaging of cell penetration of biotinylated CST in A375 cells visualized by AF488 tagged streptavidin. The nuclei are stained with Hoechst. Magnification 60X. Scale bar-5um.(**C&D**) Cell viability of mouse embryonic fibroblast (MEF) and mouse melanoma cell line B16F10 by CCK-8 assay using different concentrations of CST (0.5 μM, 1 μM, 2 μM, and 5 μM) versus vehicle control for 0, 24 ,48 and 72 hours. (**E&F)** Cell viability by CCK-8 assay on normal human skin fibroblast and melanoma cell lines A375 ,SKMEL28 in response to different concentrations of CST (0.5 μM, 1 μM, 2 μM, and 5 μM) for 0, 24, 48 and 72 hours. For all cck-8 assay (n=4). (**G&H)** Colony formation assay and its quantitative analysis in B16F10 mouse melanoma cells. **(I&J**) Colony formation and its quantitative analysis in A375 human melanoma cell (n=3). (**K&L**) Transwell migration assay in CST versus vehicle-treated condition and its quantitative analysis in B16F10 cells. (**M-N)** Transwell migration assay in CST versus vehicle-treated condition along with quantitative analysis in A375 human melanoma cells (n=3). These bright field images were captured in Keyence microscope at 10X magnification. Scale bar: 100 μm. (**O-Q)** Caspase3/7 Assay in CST versus vehicle-treated condition in B16F10, A375 and SKMEL28 cells (n=5). Data were presented as Mean ± SEM. Two-way ANOVA followed by Dunnett’s multiple comparison test was used to analyze cell viability assay data. Welch’s t-test was used to analyze colony formation, transwell migration and Caspase3/7 assay data. *p ≤ 0.05, **p ≤ 0.01, ***p ≤ 0.001, and ****p ≤ 0.0001.

To further our preliminary observations on CST’s melanoma-suppressive role, we performed experiments in established mouse and human melanoma cell lines and their normal counterparts as shown in schematic (Fig. 2A). We treated human melanoma cell line A375 with biotinylated CST for 45 minutes and added AF488 tagged streptavidin to visualize CST localization. Confocal imaging revealed CST uptake into A375 cells (Fig. 2B). We next performed cell viability assay in mouse melanoma cell line B16F10 and human melanoma cell lines A375, SKMEL28 with increasing concentration of CST (0.5 μM, 1 μM, 2 μM, and 5 μM) for 72 hours. While there was no significant reduction in cell viability of normal mouse fibroblast or human skin fibroblast cell lines (Fig. 2C&E) we recorded a decline in cell viability of the melanoma cell lines (Fig. 2D&F, **Supplementary Fig. S2A**), thus indicating that CST targets the cancer cells sparing the normal ones. Based on the maximal effects, a concentration of 2 μM was used for subsequent assays.

Phase-contrast microscopy revealed increased cell death in A375 cells treated with CST for 24 hours (**Supplementary Fig. S2B**). Colony-formation assays showed that CST ablated clonogenic potential of both human and mouse melanoma cells (Fig. 2G–J; **Supplementary Fig. S2C&D**). Because cell migration is a hallmark of metastatic potential, we evaluated migration using transwell and wound-healing assays. CST significantly impaired migration in all melanoma lines compared with controls (Fig. 2K–N; **Supplementary Fig. S2E-H**). Increased caspase-3/7 activity in CST-treated melanoma cells (Fig. 2O–Q) further confirmed apoptotic induction. Collectively, CST inhibits melanoma cell survival, proliferation, and metastatic potential.

### 3.3 Catestatin reduces melanoma tumor burden *in vivo*

**Figure 3:**
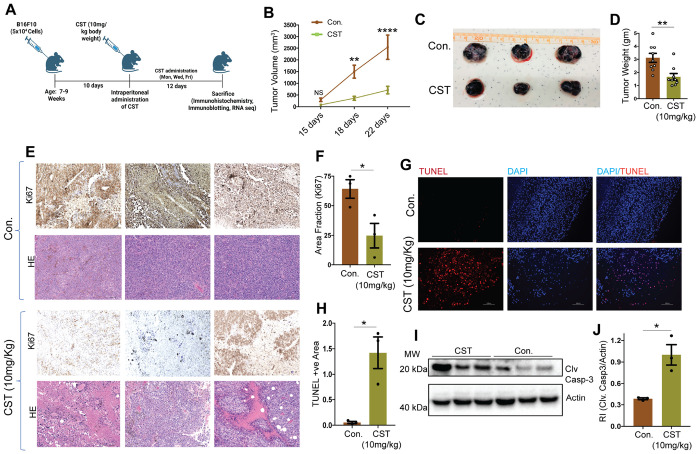
*In vivo* melanoma tumor is reduced upon CST treatment (**A**) Schematic showing injection of 5*10^^4^ B16F10 cells to 7-9 weeks old C57BL/6 mice and administration of CST (10 mg/kg) thrice a week till sacrifice. (**B)** Tumor growth kinetics in B16F10 derived tumors treated with CST (10mg/kg) and vehicular control (n=9). (**C)** Post-harvesting images of Control and CST-treated tumors. (**D)** Bar graph showing changes in tumor weight in response to control or treatment with CST (n=9). (**E)** Immunohistochemistry images showing levels of proliferation marker Ki-67 in Control and CST treated mice tumors. Hematoxylin and eosin staining reveal the tissue structures of the tumors. Images were captured in brightfield in Keyence microscope at a magnification of 20X. Scale bar: 50 μm. (**F)** Bar graph showing area fraction of Ki-67 staining in control and CST-treated tumors (n=3). (**G)** TUNEL assay performed on control and CST treated tumor sections. The TUNEL positive cells stain red and the nuclei stain blue. Images were captured in a Keyence microscope at magnification of 10X. Scale bar: 100 μm. (**H**) The TUNEL-positive area of different tumors are represented in the graph (n=3). (**I)** Representative western blot of cleaved caspase3 as compared to actin levels in mice after treatments with control or CST treated tumors. (**J**) Bar graph showing densitometric analysis of I (n=3). Data were presented as Mean ± SEM and analyzed by 2-way ANOVA followed by Tukey’s multiple comparison test (tumor growth kinetics) or by Welch’s t-test. *p ≤ 0.05, **p ≤ 0.01, and ****p ≤ 0.001.

To assess CST efficacy *in vivo*, C57BL/6 mice were subcutaneously injected with 5 × 10^4^ B16F10 cells and treated intraperitoneally with CST (10 mg/kg, three times per week) (Fig. 3A). Our invitro plasma stability results revealed that percent remaining declined mono-exponentially over 24 h. Linear regression of In (% remaining) versus time yielded a slope of − 0.1246 h^−1^ and an intercept of 4.0108, corresponding to an apparent first-order rate constant k = 0.1246 h^−1^ and a half-life t_1/2_ = 5.56 h. Model fit was strong (R^2^ = 0.971). These results indicate moderate plasma stability for the peptide under the assay conditions, with ~ 50% of intact material remaining at ~ 5.6 h (**Supplementary Fig. S3A**). We found the following in vivo plasma pharmacokinetic values: *C*_max_ (maximum concentration): 160.3 ng/ml; *T*_max_ (time to reach Cmax): 0.25 hour; *t*_1/2_ (half-life): 1.49 hour (**Supplementary Fig. S3B**). CST treatment significantly suppressed tumor growth compared with vehicle controls (Fig. 3B) as revealed from the tumor volume kinetics. Tumor weight also decreased in CST-treated mice (Fig. 3C&D) No major changes in body weight or liver histology were observed (**Supplementary Fig. S3C&D**), indicating systemic tolerability. IHC staining revealed reduced Ki-67 expression, indicating diminished proliferation (Fig. 3E&F). Hematoxylin and eosin staining showed reduced cellular density (Fig. 3E), and TUNEL assay demonstrated increased apoptotic nuclei in CST-treated tumors (Fig. 3G&H). Western blot analysis confirmed elevated cleaved caspase-3 levels (Fig. 3I&J). Thus, CST treatment significantly curtails melanoma growth *in vivo* without observable toxicity.

### 3.4 Molecular mechanisms underlying CST-mediated melanoma regression

**Figure 4:**
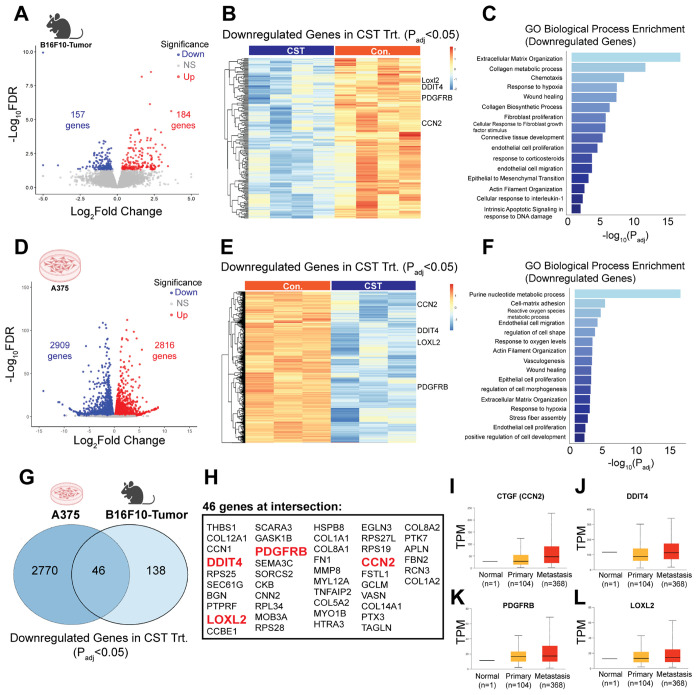
Molecular mechanisms driving CST function in mouse tumor and human melanoma cell line. (**A**) Volcano plot of differential regulation of genes upon CST treatment. (**B)** Heat map showing all downregulated genes with padj <0.05 with emphasis to *CCN2*, *LOXL2*, *DDIT4*, *PDGFRB*, and FN1 genes (n=4, each group) (**C**) Gene ontology (GO) analysis of enriched downregulated pathways emphasizing on the previously mentioned genes. (**D)**Volcano plot of differentially expressed genes upon CST treatment in A375 human melanoma cell line. (**E)** Heatmap of downregulated genes with padj <0.05 (n=3, each group) (**F)** GO analysis of enriched downregulated pathways upon CST treatment in A375 cells. (**G)** Venn Diagram showing common downregulated genes in B16F10 derived mouse tumor and A375 cell line. (**H)**List of the 46 common genes in the Venn Diagram with emphasis on *CCN2*, *LOXL2*, *DDIT4*, *PDGFRB*, and *FN1*. (**I-L)** Expression levels of *CCN2*, *DDIT4*, *PDGRB*and *LOXL2* from TCGA database in primary melanoma vs metastasis as analysed in UALCAN database.

To elucidate mechanisms of CST-induced tumor suppression, bulk RNA sequencing was performed on control and CST-treated B16F10 tumors. Differential expression analysis revealed widespread transcriptional modulation (Fig. 4A) with 184 upregulated and 157 downregulated genes upon CST treatment *in vivo*. Gene-ontology enrichment of upregulated (**Supplementary Fig. S4A&B**) and downregulated transcripts were mapped and when emphasized on the downregulated pathways they were linked to *extracellular matrix (ECM) organization, collagen metabolism, response to hypoxia*, and *epithelial-to-mesenchymal transition (EMT)* (Fig. 4B&C) - processes that facilitate melanoma progression (32–34).

Similarly, transcriptomic analyses of CST-treated A375 cells showed 2816 upregulated and 2909 downregulated genes (Fig. 4D) The differentially expressed genes (**Supplementary Fig. S4C&D**) were mapped and the downregulated pathways related to *cell-substrate adhesion*, *reactive oxygen species metabolism*, and *hypoxia response* (Fig. 4E-F) were also observed in human melanoma A375 cell line. Comparative analysis of the mouse and human cell line datasets revealed 46 commonly downregulated genes (Fig. 4G&H), including *CCN2*(Cellular Communication Network factor2), *LOXL2* (lysyl oxidase like-2), *DDIT4* (DNA damage inducible transcript 4) and *PDGFRB* (Platelet derived growth factor B), all previously implicated in melanoma aggressiveness (35–38). Consistent with these findings, TCGA-SKCM analyses via UALCAN confirmed elevated expression of these genes in advanced melanoma stages (Fig. 4I–L, **Supplementary Table 2**). Together, these results suggest that CST suppresses melanoma by downregulating ECM remodeling, hypoxia, and EMT-associated signaling programs.

### 3.5 CST treatment attenuates the expression of melanoma pro-survival genes CCN2, LOXL2, DDIT4 and PDGFRB in mice melanoma tumor and human melanoma cell line

**Figure 5:**
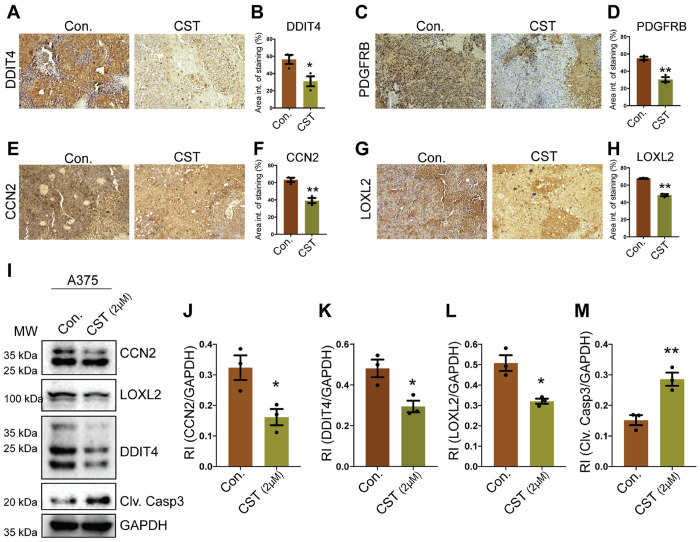
CST treatment declined expression of pro survival proteins in melanoma mice tumor and human melanoma cell line. (**A**) Immunostaining of Con and CST treated Formalin fixed paraffin embedded mice tumor tissue section for DDIT4 antigen. (**B**) Quantification of DDIT4 staining. (**C**) Immunostaining of PDGFRB in con and CST treated mice tumor section. (**D**) Quantification of PDGFRB staining. (**E&F**) Immunostaining of Tumour sections for CCN2 antigen and its quantification. (**G&H**) Immunostaining and quantification of tumour tissue sections for LOXL2 antigen.(**I**) A375 human melanoma cell line treated with CST and control and western blot of CCN2, LOXL2, DDIT4 and cleaved caspase3.(**J-M**) Quantification of CCN2, DDIT4, LOXL2, cleaved caspase3. All quantification graphs were performed on sample size(n=3) and analysed by Welch’s t-test. *p ≤ 0.05, **p ≤ 0.01, ***p ≤ 0.001, and ****p ≤ 0.0001.

The importance of genes *CCN2, LOXL2*, *DDIT4* and *PDGFRB* in melanoma progression is well established. CCN2, a connective tissue growth factor protein is expressed abundantly in melanoma and aids in metastasis by allowing cellular movement through extracellular matrix (38) LOXL2 is known to be upregulated in clinical human melanomas compared to benign nevi and often associated with metastasis and low survival of patients (39). DDIT4 on the other hand is a DNA damage inducible protein and has elevated expression in several cancers where it can promote angiogenesis and thus enhance cancer progression (39, 40). PDGFRB plays a crucial role in melanoma, and its upregulation has been often associated with drug resistance (35, 41). These genes are involved in forming new vasculature, respond to hypoxia and directly and indirectly promote EMT thereby worsening the prognosis. We further validated the expression of the proteins associated with these genes in B16F10 derived mouse melanoma tumor. Control and CST treated tissue sections were immunostained for proteins DDIT4 (Fig. 5A&B), PDGFRB (Fig. 5C&D), CCN2 (Fig. 5E&F), LOXL2 (Fig. 5G&H) and decreased expression levels of these proteins were observed in CST treated tissue sections .Quantification of these protein levels in Con and CST treated tumors revealed a significant reduction in protein expression levels. In human melanoma cell line A375 treated with CST and its control we observed diminished expression of CCN2, DDIT4, LOXL2 proteins and increased cleaved caspase 3 levels (Fig. 5I-M) thereby implicating the pro-apoptotic role of CST administration in melanoma.

### 3.6 CST suppresses Vemurafenib-resistant melanoma

**Figure 6:**
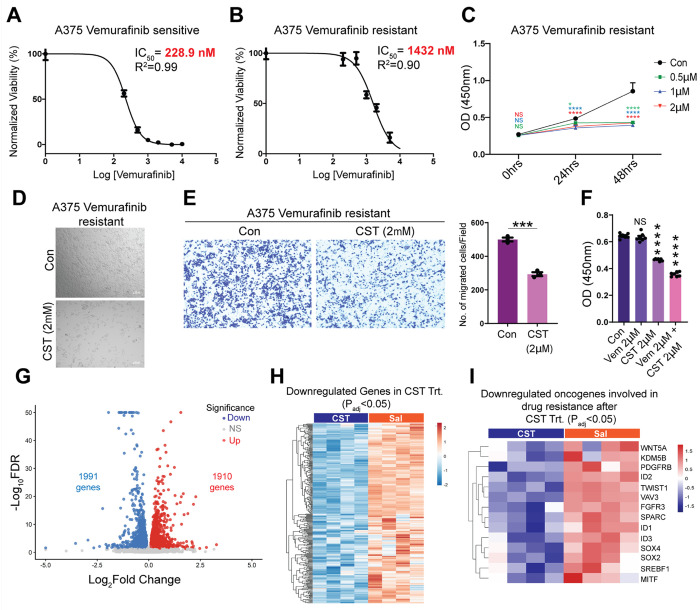
CST kills Vemurafenib resistant A375 melanoma cells by diminishing the levels of resistance associated genes. (**A**) IC_50_ of Vemurafenib in Vemurafenib-sensitive A375 cell line. (**B)** IC_50_ of Vemurafenib in Vemurafenib-resistant A375 cell line. (**C)** Dose-dependent (0.5 μM, 1 μM, 2 μM, and 5 μM) effects of CST on cell viability of Vemurafenib-resistant A375 cells after 0 ,24 and 48 hours of treatment (n=6). (**D)** Phase contrast image of control versus CST-treated Vemurafenib resistant A375. (**E)** Bar graph showing quantitative analysis of Trans well migration assay in Vemurafenib-resistant A375 cells (n=3). (**F**) Cell viability of Vemurafenib resistant A375 cells treated with Vemurafenib, CST and Vemurafenib along with CST and control for 24 hours. (**G**) Volcano plot of differentially expressed genes upon CST treatment in A375 resistant cell (n=4, each group) **(H)** Heatmap showing downregulated genes upon CST treatment with padj <0.05. (**I)** Heatmap of downregulated oncogenes with known link to resistance towards standard melanoma treatment including *WNT5A*, *KDM5B*, *PDGFRB*, *ID1*, *ID2*, *ID3*, *TWIST1*, *VAV3*, *FGFR3*, *SPARC*, *SOX2*, *SOX4*, *SREBP1*, and *MITF*. Cell viability assay graphs were analysed using 2-way ANOVA followed by Dunnett’s multiple comparison test and Welch’s t test for analysis of the transwell migration data. Cell viability assay graph of Vemurafenib, CST treatment alone or in combination was analysed using 1-way ANOVA. *p ≤ 0.05, **p ≤ 0.01, and ***p ≤ 0.001.

Resistance to BRAF inhibitors such as Vemurafenib remains a major clinical challenge. Notably, the patient-derived melanoma line 156681 (detailed in **Supplementary Table 1**), isolated from a Vemurafenib-treated non-responder, displayed enhanced CST sensitivity with increased cell death as observed previously (Fig. 1). This prompted us to check CST’s anti-cancerous potential in Vemurafenib resistant cells. To validate this observation, we generated a Vemurafenib-resistant A375 cell line. The IC_50_ for resistant cells (1432 nM) was > 6-fold higher than that of parental cells (228.9 nM) (Fig. 6A&B). CST treatment (0.5 μM, 1 μM, and 2 μM) markedly reduced viability of resistant A375 cells (Fig. 6C) and induced extensive cytopathic changes (Fig. 6D). Transwell migration assays confirmed diminished migration following CST exposure (Fig. 6E). To elucidate whether CST could overcome Vemurafenib resistance we treated the resistant cells with Vemurafenib(2uM), CST (2 μM), their combination and control. After 48 hours Vemurafenib alone had little effect on cell viability while CST alone and in combination with Vemurafenib led to a substantial decrease in cell viability of the A375 resistant cells indicating that CST can reverse the acquired resistance (Fig. 6F). To further into the molecular mechanism of these observed effects, transcriptomics was performed in control and CST-treated resistant A375 cells.

Transcriptomic analysis of resistant cells treated with CST revealed extensive transcriptional reprogramming (Fig. 6G&H, **Supplementary Fig. S5A&B**) with 1990 upregulated and 1991 downregulated genes, including downregulation of *WNT5A* (42), *PDGFRB* (43), *ID1–3* (44–46), *FGFR3* (47), *SREBF1* (48), *VAV3* (49), *KDM5B* (50–52), *TWIST1* (53), *SOX2* (54), *SOX4* (55), *MITF* (56), and *SPARC* (57) (Fig. 6I) - genes associated with resistance and metastasis. The suppression of these genes that are actively involved in conferring resistance to known treatment regimens underscores the therapeutic potential of CST in overcoming treatment-resistant melanoma.

## 4. DISCUSSION

Melanoma incidence continues to rise worldwide, with an estimated 331,647 new cases and 58,645 deaths reported in 2022 (GLOBOCAN 2022) (58). In the United States alone, over 104,000 new invasive melanoma cases are projected for 2025 (https://www.curemelanoma.org/blog/over-104-000-americans-estimated-to-bediagnosed-with-invasive-melanoma-in-2025). Despite breakthroughs in targeted and immune-based therapies, recurrence and therapy resistance remain formidable clinical barriers (59). This challenge underscores the need for novel therapies capable of curbing melanoma and overcoming resistance.

To our knowledge this study provides the first evidence linking CST to carcinogenesis and demonstrate its previously unrecognized role in melanoma, thereby positioning it as a novel therapeutic agent. Peptide-based therapeutics are underexplored in melanoma (60–63), although they offer inherent advantages, including biocompatibility, low immunogenicity, and ease of modification. CST, known for its therapeutic potential (16, 22, 11), emerged as a rational candidate owing to its regulatory roles in inflammation, oxidative stress, and metabolic homeostasis - processes intimately linked to onco-pathobiology.

CST expression decreases with melanoma progression, suggesting its loss promotes tumor progression. Restoration of CST in patient-derived melanoma cells elicit marked cytotoxicity and apoptosis in lines with distinct molecular features - K06184 (wild-type BRAF, brain metastasis) and 156681 (BRAF^V600E, Vemurafenib-non-responder) and 128128 (treatment naïve, metastatic melanoma) - highlighting its broad anti-cancer efficacy across genotypes.

CST exerted potent anti-proliferative and anti-migratory effects. In both mouse (B16F10) and human (A375, SKMEL28) melanoma cell lines, CST treatment reduced cell viability, colony formation, and transwell migration. *In vivo*, CST administration attenuated tumor growth in B16F10 melanoma-bearing C57BL/6 mice, with concomitant reductions in Ki-67 and elevations in TUNEL and cleaved-caspase-3 signals. The absence of changes in body weight or hepatic histology confirmed that CST is well tolerated and systemically non-toxic.

To define CST-driven molecular alterations, transcriptomic analyses identified downregulated gene clusters enriched in pathways controlling *extracellular-matrix organization*, *collagen metabolism*, *hypoxia adaptation*, and *epithelial-to-mesenchymal transition (EMT)* - all central to melanoma invasion and metastasis. CST significantly suppressed *CCN2, LOXL2, DDIT4*, and *FN1*, key drivers of ECM remodeling and angiogenesis, indicating that CST disrupts the structural and metabolic axes sustaining tumor progression.

Given the prevalence of therapeutic resistance, particularly to BRAF/MEK inhibitors, we investigated any potential role of CST in Vemurafenib-resistant A375 cells. CST markedly reduced cell viability and migration in these resistant cells, accompanied by downregulation of multiple resistance-associated genes - including *FGFR3* (47, 64), *ID1/2/3* (44, 65), *PDGFRB*, *SREBF1* (48, 66), and *MITF* (67). These targets are central mediators of adaptive survival in BRAF^V600E melanoma, suggesting that CST not only restrains tumor growth but may re-sensitize resistant cells to existing therapies.

Overall, our findings delineate that CST suppresses melanoma growth and resistance with a favorable safety profile. Although CST has been variably reported to promote angiogenesis in other contexts (28, 68), our *in-vivo* and transcriptomic data consistently support an anti-angiogenic and anti-proliferative role in melanoma.

As CST emerges as novel modulator of carcinogenesis, further detailed mechanistic studies are warranted to fully establish its therapeutic potential. In parallel, peptidomimetic optimization of CST to enhance stability, half-life, and tumor bioavailability will enhance its therapeutic potential. Combination therapy paradigms integrating CST with BRAF/MEK inhibitors or immune checkpoint blockade warrant exploration to achieve synergistic efficacy. Moreover, since CgA yields several bioactive fragments - PST, Vasostatin I/II, WE14, and Serpinin (14) - systematic studies of their processing and interactions in melanoma may reveal complementary or cooperative functions.

In conclusion, this study provides compelling evidence that Catestatin represents a novel, safe, and effective therapeutic modality for both treatment-naïve and drug-resistant melanoma. By simultaneously targeting tumor growth, metastatic potential, and resistance pathways, CST holds significant translational promise as the foundation for a new class of peptide-based anti-cancer therapies.

## Supplementary Material

This is a list of supplementary files associated with this preprint. Click to download.

• LegendstoSupplementaryFigures.docx

• SupplementaryTable1.docx

• SupplementaryFigure4.tif

• SupplementaryFigure2.tif

• SupplementaryFigure3.tif

• SupplementaryTable2.docx

• SupplementaryFigure5.tif

• OriginalWesternBlots.tif

• SupplementaryFigure1.tif

## Data Availability

Data supporting the conclusion of the current study is included in the manuscript and supplementary files. Bulk RNA sequencing data is available on **GSE312638** and the codes used for RNA Seq analysis are available on reasonable request. Uncropped images for the western blots are included in **Supplementary Fig. 6**.
